# Clinical characteristics and prognosis of paraneoplastic syndromes: a single-center cohort study in Northern China

**DOI:** 10.3389/fimmu.2025.1715164

**Published:** 2026-01-07

**Authors:** Minzhe Hu, Qianchang Wang, Yuxiu Xiao, Danqing Qin, Shougang Guo, Chunjuan Wang

**Affiliations:** 1 Department of Neurology, Shandong Provincial Hospital Affiliated to Shandong First Medical University, Jinan, Shandong, China; 2 Shandong First Medical University & Shandong Academy of Medical Sciences, Jinan, Shandong, China; 3 Department of Neurology, Shandong Provincial Hospital, Shandong University, Jinan, Shandong, China

**Keywords:** clinical outcomes, encephalitis, long term follow-up, paraneoplastic syndrome, prognosis

## Abstract

**Purpose:**

To explore the clinical features, treatment, and prognosis of paraneoplastic neurological syndromes (PNS).

**Methods:**

In this retrospective cohort study, the records of 114 patients diagnosed with probable (n = 65) or definite (n = 49) PNS between July 2016 and October 2024 were analyzed. Short-term outcome was defined as the point decrease in modified Rankin Scale score from peak disease to discharge(Δmodified Rankin Scale). Long-term prognosis was determined by mortality at last follow-up. Prognostic factors were identified using logistic regression and Cox models. The impact of tumors and high-risk antibodies on survival were assessed by Kaplan–Meier curves.

**Results:**

Of the 114 patients, 65 (57.0%) were males. The median age was 63 years. Muscle weakness (53.5%) was most common, followed by seizures and altered consciousness. Associated tumors occurred in 66.7% of patients, mainly lung (65.8%) and breast cancer (9.2%). Antibodies were detected in 79.8%, including (single and multiple antibody types) anti-GABA_B_R (24.2%), anti-Hu (19.8%), and anti-SOX1 (19.8%). Multiple antibodies were detected in 18.4%, including anti-Hu plus anti-SOX1 (19.0%), anti-SOX1 plus anti-GABA_B_R (14.3%), and others. Independent factors associated with short-term favorable outcome (Δmodified Rankin Scale ≥1) were age < 65 years(OR = 3.41, 95% CI: 1.45–7.98, P = 0.005), CNS involvement (OR = 2.46, 95% CI: 1.05–5.80, P = 0.039), and immunotherapy (OR = 5.12, 95% CI: 1.70–15.42, P = 0.004). The median survival was 32 months (IQR, 12–106), the 3-year survival rate was 45.8%. SCLC (HR = 3.04, 95% CI: 1.71–5.41, P < 0.001) and high-risk antibodies (HR = 2.06, 95% CI: 1.17–3.62, P = 0.012) were independently associated with higher mortality.

**Conclusions:**

Age < 65 years, CNS involvement and immunotherapy are relevant to favorable short-term outcome. SCLC and high-risk antibodies are adverse factors of long-term survival in PNS.

## 1 Introduction

Paraneoplastic neurological syndromes (PNS) are a group of immune-mediated disorders closely associated with malignancies, characterized by a diverse array of complex clinical manifestations (1, 2). These syndromes may affect both the central and peripheral nervous systems and are not due to direct tumor invasion, compression, or metastasis. Additionally, PNS cannot be explained by complications arising directly from the tumor itself, as it results from an immune response triggered by the malignancy (1–4).

Patients with PNS often present specific onconeural antibodies in both serum and/or cerebrospinal fluid, including anti-Yo, anti-Hu, anti-CV2, anti-Ri, anti-Amphiphysin, anti-Ma2, anti-GAD65 antibodies and so on. These antibodies mainly target intracellular neuronal antigens, typically located in the nucleus, cytoplasm, or synaptic structures. Collectively, the presence of these antibodies and their specific antigenic targets strongly suggest that autoimmune mechanisms are pivotal in the pathogenesis of PNS (5, 6).

The management of PNS involves not only the treatment of the underlying malignancy but also the application of additional immunological interventions. These immunomodulatory therapies predominantly include corticosteroids, intravenous immunoglobulins, plasma exchange, and rituximab, among others (7, 8). The prognosis for PNS is generally regarded as unfavorable, influenced by both the underlying malignancy and the severity of the associated neurological manifestations (9, 10). Despite the implementation of standardized treatment protocols, the progression of PNS remains highly variable, making it challenging to accurately predict prognosis. Here in, we aimed to investigate the clinical characteristics, treatment strategies, and prognostic factors associated with PNS.

## 2 Methods

### 2.1 Patient selection

Data of the patients who were hospitalized in the Department of Neurology of Shandong Provincial Hospital Affiliated to Shandong First Medical University between July 2016 and October 2024 were collected. The following inclusion criteria applied: (1) age at onset ≥ 18 years, (2) clinical presentation consistent with a paraneoplastic disease at presentation, including encephalomyelitis, rapidly progressive cerebellar syndrome, limbic encephalitis, sensory neuropathy, lambert-eaton myasthenic syndrome, brainstem encephalitis, isolated myelopathy, polyradiculoneuropathy, (3) positive testing for paraneoplastic antibodies or a definitive tumor diagnosis during hospitalization or follow-up, and (4)conforming to diagnosis criteria of definite or probable PNS based on the 2021 diagnostic criteria for PNS, in which ≥ 8 points indicate definite PNS and 6–7 points indicate probable PNS (5). Exclusion criteria were as follows: (1) lack of follow-up information, (2) incomplete clinical data, and (3) follow-up duration less than 6 months. The paraneoplastic antibodies included Yo, Hu, Ri, Ma2, CV2, SOX1, Amphiphysin, GAD65, Tr antibodies (immunoblotting and immunofluorescence) and CASPR2, AMPAR, LGI1, VGCC and GABA_B_R antibodies (fixed-CBA and immunofluorescence). The diagnosis of PNS was considered at the time the oncological antibody test result was obtained. Cancer diagnosis was dated to the biopsy procedure in cases with pathological confirmation. In cases where a biopsy was not performed, the diagnosis was instead determined by the timing of imaging examinations. All patients were independently reviewed by two neurologists, and any discrepancies in the assessments were resolved through consensus.

### 2.2 Data collection

We collected data on patient demographics, clinical manifestations, imaging findings, electrophysiological results, associated malignancies (when present), and identified autoantibodies (if any), along with treatment regimens and neurological outcomes. All patients underwent a minimum follow-up period of six months. Neurological function was assessed using the modified Rankin Scale (mRS) by two experienced neurologists at three key time points: disease peak, discharge and final follow-up. The last recorded mRS score served as the endpoint for neurological assessment. Information on patient survival (including dates of death) was recorded to assess long-term outcomes.

### 2.3 Clinical outcomes

Short-term efficacy was evaluated by ΔmRS, defined as the difference in mRS scores between discharge and the disease peak. Favorable short outcome was defined as an improvement of ΔmRS ≥ 1 point. Long-term prognosis was assessed based on all-cause mortality at the final follow-up, reflecting the patient’s overall survival status.

### 2.4 Statistical analysis

Patient clinical characteristics were summarized by descriptive statistics. Quantitative data with a normal distribution were expressed as mean ± standard deviation; skewed data were presented as median(IQR). Categorical data were presented as count and percentage (n [%]). Univariate logistic regression analysis was performed to identify factors associated with the efficacy of immunotherapy at discharge. Univariate Cox proportional hazards models were used to assess prognostic factors related to mortality. Variables yielding with a P-value < 0.1 in the univariate analyses, as well as those deemed clinically relevant, were included in the respective multivariate logistic or Cox regression models. Kaplan-Meier survival curves were employed to evaluate the influence of cancer diagnosis, cancer type, and high-risk antibodies on survival time. A p-value of less than 0.05 was considered statistically significant. Statistical analyses were performed with SPSS (IBM, version 27.0), and figures were created using GraphPad Prism 10 and Origin 2025. Statistical analyses were not conducted for patient characteristics according to antibody groups, as these antibodies were not mutually exclusive.

## 3 Results

### 3.1 Demographic and clinical characteristics

All 114 patients consisted of 65 males (57.0%) and 49 females (43.0%), with a median age of 63 years (IQR: 55–69), including 65 probable and 49 definite PNS cases. Among these PNS patients, myasthenia (53.5%) was the most prevalent clinical manifestation. Other common clinical features included seizures, consciousness disturbances, sensory deficits, cognitive impairment, ataxia, dizziness, dysarthria and psychiatric symptoms. Some patients also experienced headaches and diplopia. In this cohort study, nine distinct subtypes of PNS were identified. The frequencies of these subtypes were as follows: encephalomyelitis (39/114, 34.2%), limbic encephalitis (20/114, 17.5%), Lambert-Eaton myasthenic syndrome (19/114, 16.7%), sensory neuronopathy (15/114, 13.2%), rapidly progressive cerebellar syndrome (8/114, 7.0%), isolated myelopathy (6/114, 5.3%), polyradiculopathy (4/114, 3.5%), brainstem encephalitis (2/114, 1.8%), and opsoclonus myoclonus (1/114, 0.9%). The clinical characteristics of patients with different subtypes of PNS are shown in **Table 1**.

**Table 1:** Characteristics of the study sample.

TALLE 1	Total(n=114)	PNS patients	Definite (n=49)
		probable (n=65)	
Demographics			
Age at symptom onset	63(55, 69)	63.0(55.5, 68.5)	62.3±9.3
Sex (male/female)	65/49	41/24	24/25
Clinical manifestations (n, %)			
Seizures	38(33.3)	20(30.8)	18(36.7)
Consciousness disturbance	40(35.1)	22(33.8)	18(36.7)
Psycho-behavioral abnormalities	11(9.6)	4(6.2)	7(14.3)
Speech disorder	22(19.3)	9(13.8)	13(26.5)
Cognitive disoeder	31(27.2)	19(29.2)	12(24.5)
Ataxia	26(22.8)	12(18.5)	14(28.6)
Sensory disorder	34(29.8)	20(30.8)	14(28.6)
Myasthenia	61(53.5)	32(49.2)	29(59.2)
Headache	7(6.1)	4(6.2)	3(6.1)
Dizziness	21(18.4)	10(15.4)	11(22.4)
Diplopia	7(6.1)	3(4.6)	4(8.2)
CSF			
Number of cases with elevated cerebrospinal fluid pressurel (range, mmH2O)	9(60-300)	4(60-285)	5(85-300)
Number of cases with increased protein level (range, mg/L)	35(220-2070)	21(240-1290)	14(220-2070)
Number of cases with cerebrospinal fluid pleocytosis (>5/mm3) (range, 10^6^ cells/L)	30(0-118)	13(0-48)	17(0-118)
Cases with abnormalities in brain magnetic resonance imaging and spinal cord magnetic resonance imaging(n)			
Frontal bobe	7	3	4
Parietal lobe	5	2	3
Temporal lobe	4	3	1
Occipital lobe	3	2	1
Basal ganglia	3	0	3
Brain stem	3	2	1
Cerebellum	2	2	0
Insular lobe	14	9	6
Cervical cord	5	5	0
Thoracic spinal cord	1	1	0
Positive antibody(n)			
VGCC	13	3	10
GABA_B_R	22	6	16
Hu	18	7	11
SOX1	18	4	14
Amphiphysin	7	4	3
CV2	8	2	6
Ma2	4	3	1
Ri	5	4	1
GAD65	1	0	1
Tr	4	4	0
AMPAR	1	0	1
LGL1	3	3	0
CASPR2	1	0	1
Yo	9	6	3
Cases with abnormal electroencephalogram (n)			
Total with abnormal findings	34	19	15
Epileptiform discharges	24	16	8
Slow waves	26	16	10
Treatments (n)			
No immunotherapy	24	19	5
Only steroids	60	28	32
Steroids + IVIG	28	16	12
Steroids + plasma exchange	2	2	0
Immunosuppressant	11	7	4
mRS			
Pre-treatment mRS score(Median,range)	3(1,5)	2(1,5)	3(1,5)
mRS score after first-line immunotherapy(range)	2(0,5)	2(0,5)	2(0,5)
Cancer			
SCLC	50	13	37
Breast Cancer	7	4	3
Thyroid Cancer	6	3	3
Endometrial cancer	1	0	1
Pancreatic Cancer	1	0	1
Vulvar Cancer	1	0	1
Low-grade Glioma (Intracranial)	1	0	1
Ovarian Cancer	3	1	2
Thymoma	2	1	1
Cervical cancer	1	1	0
Hodgkin lymphoma	1	1	0
Ampullary cancer	1	1	0
Testicular cancer	1	1	0
Cancer treatment			
Radiotherapy+chemotherapy	15	3	12
Surgical treatment	20	13	7
Chemotherapy	7	3	4
Surgical treatment + radiotherapy + chemotherapy	3	1	2
Surgical treatment + chemotherapy	4	3	1
Surgical treatment + radiotherapy	1	1	0

Statistics presented as median [Minimum, Maximum], Abbreviations: CSF, cerebrospinal fluid; MRI, magnetic resonance imaging; SCLC, small cell lung cancer; mRS, modified Rankin Scale; IVIG, intravenous immunoglobulin;VGCC,Voltage-Gated Calcium Channel; GABA_B_R, γ-Aminobutyric Acid B ;SOX-1,Sex-determining Region Y-Box 1;GAD65,Glutamic Acid Decarboxylase 65; NMDAR, N-methyl D-aspartate receptor; MOG, Myelin Oligodendrocyte Glycoprotein; LGI1, leucine-rich glioma inactivated 1.

In terms of tumor screening, 76 patients (76/114, 66.7%) were diagnosed with associated malignancies. The most common malignancy was lung cancer (50/76, 65.8%), followed by breast cancer (7/76, 9.2%) and thyroid cancer (6/76, 7.9%). Other malignancies included vulvar cancer, low-grade gliomas, pancreatic cancer, ovarian cancer, uterine cancer, duodenal ampullary cancer, Hodgkin lymphoma, testicular cancer, and thymoma (**Table 1**). Among the 76 patients with malignancies, 50 (65.8%) received tumor-directed therapy. Treatments included surgery (28/50, 56.0%), chemotherapy (29/50, 58.0%), and radiotherapy (19/50, 38.0%). One patient additionally received immune checkpoint inhibitor therapy. Etoposide–platinum regimens were most frequently used for small-cell lung cancer, whereas anthracycline- or taxane-based chemotherapy predominated in breast cancer. A total of 26 patients (26/76, 34.2%) developed neurological symptoms after treatment for their malignancy. The median time from cancer treatment to the onset of neurological symptoms was 24 months (range: 2 months to 10 years). Additionally, 50 patients (50/76, 65.8%) presented with neurological symptoms before tumor discovery. The time interval between the onset and tumor discovery ranged from 10 days to 6 months, with a mean of 2 months. The remaining 38 patients, followed for a period of 6 to 71 months, did not develop tumors during the follow-up period.

### 3.2 Antibody distribution

All 114 patients were screened for paraneoplastic antibodies. Among them, 58.8% (67/114) underwent screening for both serum and cerebrospinal fluid (CSF), while the remaining patients were screened for serum only. Only 79.8% (91/114) of the patients tested positive for paraneoplastic antibodies. Of the 91 patients, the most common antibody subtype was anti-GABA_B_R (22 cases), followed by anti-Hu (18 cases), anti-SOX1 (18 cases), and anti-VGCC (13 cases). Fewer than 10 cases were anti-Yo, anti-Amphiphysin, anti-CV2, anti-Tr, anti-Ri, anti-Ma2, anti-LGl1, anti-CASPR2, anti-AMPA, or anti-GAD65.

**Figure 1:**
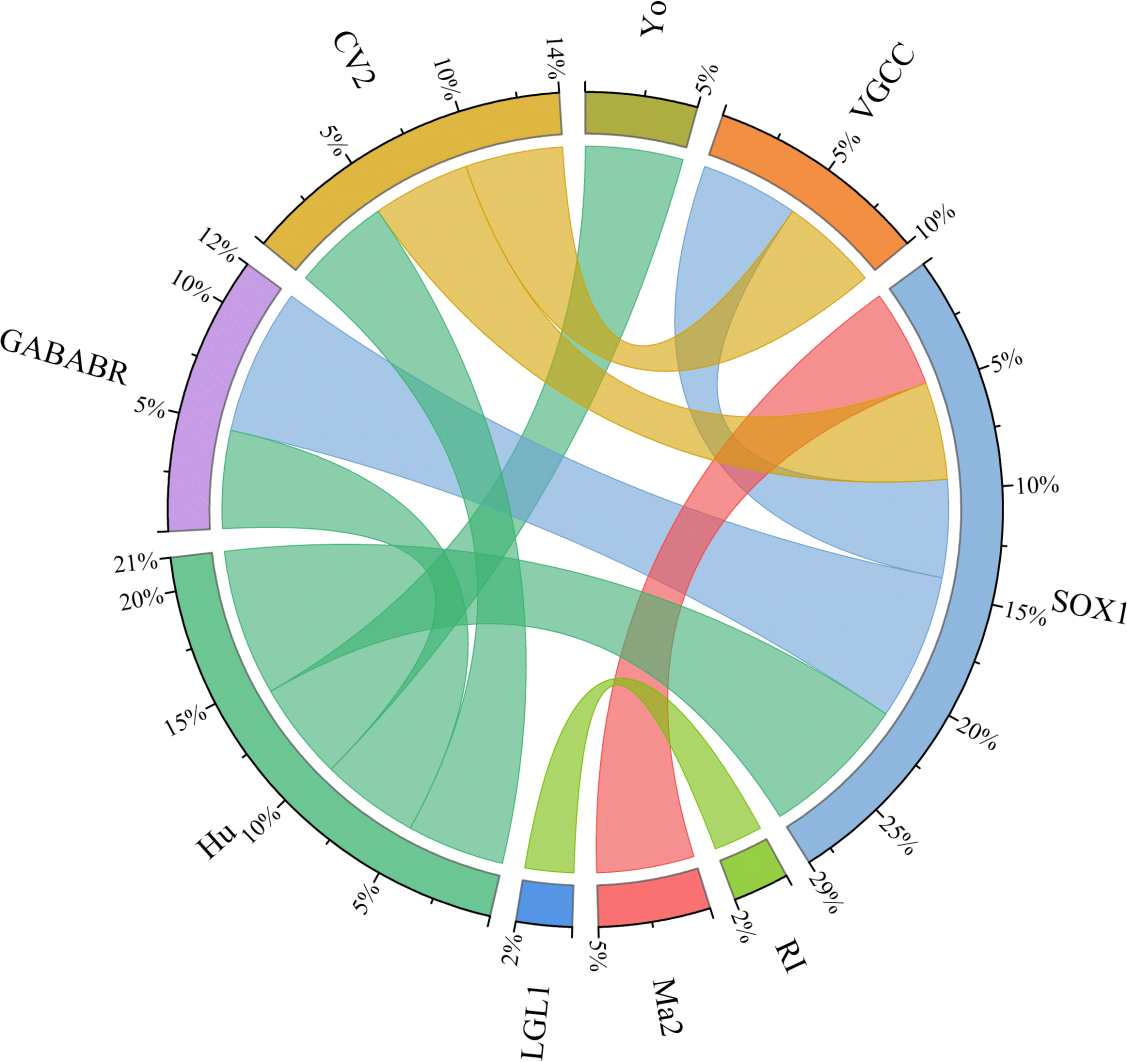
Chord diagram depicting the co-occurrence of antibodies in patients with PNS. Arc size indicates the number of patients with both antibodies.

**Figure 2:**
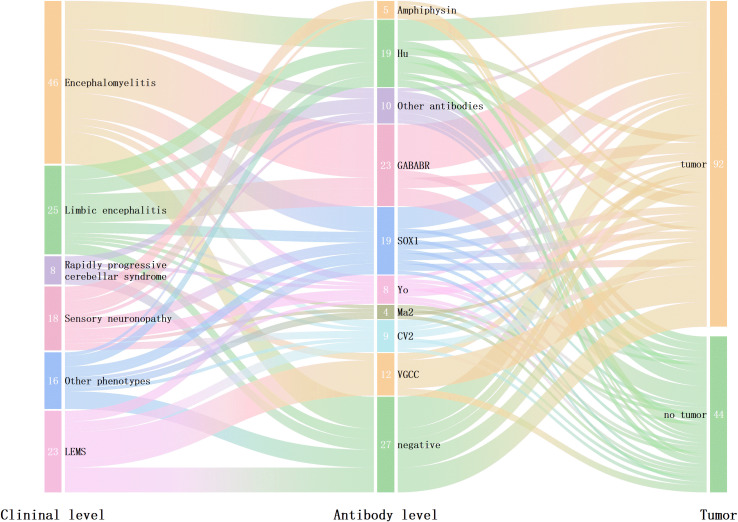
The relationship among clinical manifestations, antibodies, and the presence of tumors in patients with PNS. The left column illustrates the distribution of neurological syndromes, the middle column shows the distribution of detected antibodies, and the right column illustrates the associated tumors.

In this cohort study, 21 patients were identified with coexisting multiple anti-neural antibodies, with the most common combination being the coexistence of anti-Hu and anti-SOX1 antibodies, observed in 4 patients. In detail, 3 patients had coexistent anti-SOX1 antibody and anti-GABA_B_R, 2 had coexistent anti-Hu and anti-CV2, 2 had coexistent anti-SOX1 and anti-CV2, 2 patients had coexistent anti-SOX1 and anti-Ma2, 2 patients had coexistent anti-SOX1 and anti-VGCC, 2 patients had coexistent anti-Hu and anti-GABA_B_R, and 2 patients had coexistent anti-CV2 and anti-VGCC, 1 patients had coexistent anti-Ri and anti-LGl, and 1 patients had coexistent anti-Hu and anti-Yo. Anti-neural antibodies are frequently observed in systemic lupus erythematosus. However, no patient in our cohort had concomitant SLE (**Figure 1**). The relationships among clinical phenotypes, paraneoplastic antibodies, and tumor presence are illustrated in **Figure 2**.

### 3.3 Short-term prognostic factors

Multivariable logistic regression was applied to evaluate the relationship between explanatory variables and a good short-term outcome (ΔmRS ≥ 1) in PNS. Logistic regression analysis identified age < 65 years (OR = 3.41, 95% CI: 1.45–7.98, P = 0.005), receipt of immunotherapy (OR = 5.12, 95% CI: 1.70–15.42, P = 0.004), and presence of CNS phenotypes (OR = 2.46, 95% CI: 1.05–5.80, P = 0.039) were independently associated with favorable therapeutic outcomes(**Table 2**, **Supplementary Figure S1**).

**Table 2:** Univariate and multivariate logistic regression of short-term prognostic factors associated with ΔmRS≥1.

Variables	Univariate analysis		Multivariate analysis	
	OR(95%CI)	p-value	OR(95%CI)	p-value
Age<65yr	2.50(1.18~5.33)	**0.017**	3.41(1.45~7.98)	**0.005**
Male	0.73(0.35~1.53)	0.403	0.48(0.21~1.13)	0.092
Cancer	0.53(0.24~1.17)	0.114		
SCLC	0.96(0.46~2.01)	0.905		
CNS	2.16(1.01~4.62)	**0.048**	2.46(1.05~5.80)	**0.039**
Isolated myelopathy	0.43(0.08~2.45)	0.343		
Rapidly progressive cerebellar syndrome	0.89(0.21~3.76)	0.877		
Limbic encephalitis	0.88(0.34~2.31)	0.795		
Encephalomyelitis	1.48(0.68~3.23)	0.329		
Immunotherapy	4.50(1.63~12.43)	**0.004**	5.12(1.70~15.42)	**0.004**
Only steroids	1.62(0.77~3.41)	0.2		
Steroids and other first-line therapies	1.81(0.77~4.27)	0.174		
High-risk antibodies	0.62(0.30~1.30)	0.206		
Multiple antibodies	1.67(0.66~4.20)	0.279		

Bolded values indicate significant P-value (<0.05). Abbreviations: CNS, central nervous system; SCLC, small cell lung cancer; CI, confidence interval; OR, odds ratio.

### 3.4 Long-term prognostic factors

**Figure 3:**
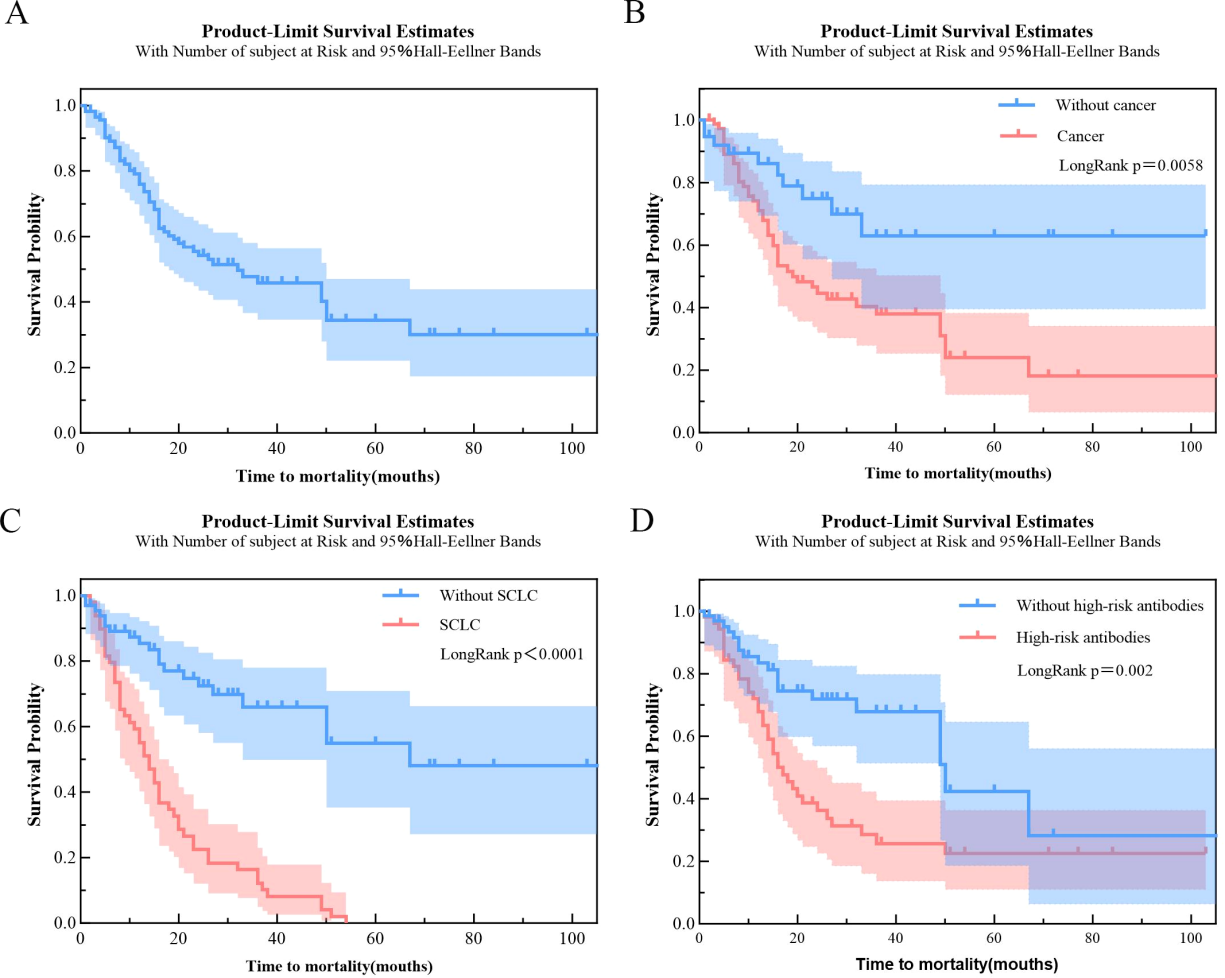
Kaplan–Meier survival curves for time to mortality: **(A)** overall cohort; **(B)** stratified by cancer status (yes vs. no); **(C)** stratified by SCLC (yes vs. no); **(D)** stratified by high-risk antibodies (yes vs. no).

The study population had a median survival of 32 months from symptom onset (IQR 12–106). At 3 years, the survival probability was 45.8% (**Figure 3A**). No association was observed between age at onset and survival. SCLC was an independent risk factor for mortality(HR = 3.04, 95% CI = 1.71–5.41, P<0.001). Additionally, high-risk antibodies(HR = 2.06, 95% CI: 1.17–3.62, P = 0.012) were independently associated with a higher risk of death (**Table 3**). Three-year survival was 37.9% in patients with cancer compared to 62.9% in those without (**Figure 3B**), while in patients with SCLC, it dropped to 12.2% (**Figure 3C**). Based on Kaplan–Meier curves, high-risk antibody–positive patients exhibited poorer 3-year survival than antibody–negative patients (P = 0.002) (**Figure 3D**).

**Table 3:** Cox proportional hazards analysis of factors associated with time from diagnosis to death.

Variables	Univariate analysis		Multivariate analysis	
	HR(95%CI)	p-value	HR(95%CI)	p-value
Male	1.36(0.78-2.38)	0.284		
Age<65yr	0.89(0.52-1.52)	0.666		
Cancer	2.52(1.27-5.01)	0.009*		
SCLC	3.28(1.86-5.80)	<0.001*	3.04(1.71-5.41)	<0.001*
Multiple antibodies	0.83(0.40-1.70)	0.605		
High-risk antibodies	2.33(1.33-4.08)	0.003*	2.06(1.17-3.62)	0.012*
CNS	0.94(0.55-1.60)	0.806		
Encephalomyelitis	1.96(0.55-1.70)	0.896		
Limbic encephalitis	0.70(0.33-1.49)	0.354		
Rapidly progressive cerebellar syndrome	0.49(0.12-2.02)	0.323		
Brainstem encephalitis	1.58(0.21-11.3)	0.667		
Isolated myelopathy	0.63(0.15-2.60)	0.524		
Sensory neuronopathy	1.25(0.59-2.65)	0.563		
LEMS	1.69(0.90-3.16)	0.101		
Polyradiculoneuropathy	0.56(0.08-4.04)	0.562		
Immunotherapy	1.49(0.72-3.04)	0.280		
Cancer treatment	1.02(0.60-1.75)	0.933		

Bolded values indicate significant P-value (<0.05). Abbreviations: CNS, central nervous system; SCLC, small cell lung cancer; HR, hazard ratio; CI, confidence interval; LEMS, Lambert-Eaton myasthenic syndrome

## 4 Discussion

In this retrospective cohort study, multiple determinants of clinical outcomes were recognized in patients with PNS. Specifically, age < 65 years, CNS involvement, and receipt of immunotherapy were each independently associated with improved short-term functional recovery. In contrast, SCLC and high-risk antibodies were important predictors of reduced long-term survival.

In our study, encephalomyelitis and limbic encephalitis represented the most common phenotypes, in accordance with prior reports (10). Lung carcinoma and breast cancer were the leading tumor types, consistent with previous literature (5, 11). As in prior research, SCLC was the most common lung cancer subtype linked to PNS, a highly immunogenic neuroendocrine tumor that expresses multiple neuronal antigens also found in the nervous system (11–13), thereby contributing to its frequent involvement in the pathogenesis of PNS.

The distribution of onconeural antibodies in our single-center cohort differed substantially from those reported in previous multicenter studies. While anti-Yo and anti-Hu were the most frequently detected antibodies in European cohorts (14, 15), anti-GABA_B_R was most common in our series, followed by anti-Hu and anti-SOX1. These differences may reflect advances in assay techniques, methodological variability, or regional differences in antibody prevalence (10), highlighting the need for standardized detection protocols in future research.

Multiple anti-neural antibodies were identified in 21 patients (18.4%), most frequently anti-Hu with anti-SOX1. Such coexistence may represent a broad immune response to diverse tumor antigens and could be related to tumor-driven mutagenic processes (16, 17). Hardy-Werbin et al. (18, 19) previously reported that the presence of a single autoantibody in patients with PNS was associated with more favorable clinical outcomes compared to multiple autoantibodies. In contrast, our study did not identify significant differences in either short-term efficacy or long-term prognosis between patients harboring single versus multiple autoantibodies, which may partly reflect the small sample size and heterogeneity of the cohort. Notably, our findings indicate that the presence of high-risk autoantibodies serves as a key determinant of poor long-term survival in patients with PNS. These antibodies are often associated with severe neuronal injury, aggressive malignancies, and poor responses to immunotherapy. (4, 6, 20).

Age < 65 years was associated with short-term favorable outcomes in our study population. This finding is in line with a prior study in patients with idiopathic or paraneoplastic opsoclonus–myoclonus (21), in which younger individuals were more likely to have favorable outcomes than their older counterparts.

In our study, CNS involvement was associated with short-term favorable outcomes, which contrasts with most previous reports linking central involvement to poorer prognosis in PNS patients (12, 20). This discrepancy may be partly explained by differences in antibody profiles between our cohort and those in prior studies. In our series, antibodies against cell-surface antigens were detected in 38.6% (44/114) of all patients, a higher proportion than the approximately 25–30% reported in large PNS cohorts, in which onconeural antibodies such as anti-Hu (≈39%) predominated (15, 22). Cell-surface antibody–mediated syndromes are generally more responsive to immunotherapy (23), which may have contributed to the favorable short-term outcomes observed in our patients despite CNS involvement. Further studies are necessary to validate these results and to clarify the prognostic implications of CNS involvement across different PNS subtypes.

Currently, treatment strategies for PNS primarily include tumor-directed therapy, first-line immunotherapy (intravenous immunoglobulin, corticosteroids, and plasmapheresis), and second-line immunosuppressants (such as cyclophosphamide and rituximab). In our cohort, immunotherapy was associated with short-term favorable outcomes, in line with previous studies in patients with PNS showing that early initiation of immunotherapy significantly improved prognosis (24, 25). Beyond conventional therapy, one patient underwent immune checkpoint inhibitor (ICI) therapy for cancer and had developed PNS before ICI initiation. This patient experienced neurological deterioration following treatment and subsequently died. Although anecdotal, this case underscores the potential for ICIs to amplify autoimmune activation and worsen neurological outcomes, consistent with previous reports linking ICI exposure to increased disability and mortality in PNS (12, 26, 27). Given the expanding use of ICIs in oncology, distinguishing PNS from ICI-related neurological adverse events has become clinically important. Such differentiation primarily depends on assessing the temporal relationship to ICI initiation, the presence of specific onconeural or cell-surface antibodies, tumor status, and treatment response patterns (28).

In this cohort, the 3-year overall survival rate was 45.8%, similar to that observed in earlier reports (12). As anticipated, SCLC was significantly associated with adverse outcomes, as reflected by a 3-year survival rate of only 12.2%. In contrast, no significant association with mortality was observed for other tumor types. Notably, our analysis did not reveal a significant association between tumor-directed therapy and overall survival in our series. However, previous studies have demonstrated that tumor-directed treatment is a major determinant of survival in patients with PNS. For example, in lung cancer–associated paraneoplastic limbic encephalitis, patients receiving tumor-specific therapy achieved neurological stabilization or improvement in most cases, with an overall tumor remission rate of 75% and a 2-year overall survival of approximately 74.7%, whereas immunotherapy alone had limited impact on survival (29, 30). Differences between our findings and prior reports may reflect variations in sample size, patient characteristics, tumor type distribution, and treatment strategies.

This study has several limitations. Only a portion of patients underwent CSF antibody testing, which may have affected diagnostic accuracy, particularly when differentiating PNS from metastatic disease or leptomeningeal involvement. Although validated assays were used for each antibody, the use of multiple testing platforms may have introduced some methodological variability. The retrospective single-center design and relatively limited sample size may also have led to selection bias and may restrict the generalizability of our findings. In addition, differences in the diagnostic workup and incomplete documentation of tumor-directed treatments could have contributed to bias or unmeasured confounding. Future multicenter studies with larger cohorts and standardized diagnostic and treatment protocols will be important to further validate these results and to refine prognostic assessment in PNS.

## Data Availability

The original contributions presented in the study are included in the article/**Supplementary Material**. Further inquiries can be directed to the corresponding author.

## Supplementary material

Supplementary Figure 1 Forest plot with univariate logistic regression predicting curative effect in patients at discharge. Error bars: 95% confidence interval. SCLC, small cell lung cancer; CNS, central nervous system.
