# Supplementary figures and images for: Clinical characteristics and prognosis of paraneoplastic syndromes: a single-center cohort study in Northern China
Source: Front Immunol. 2026 Jan 7;16:1715164. doi: 10.3389/fimmu.2025.1715164 (PMC12819185; doi:10.3389/fimmu.2025.1715164)

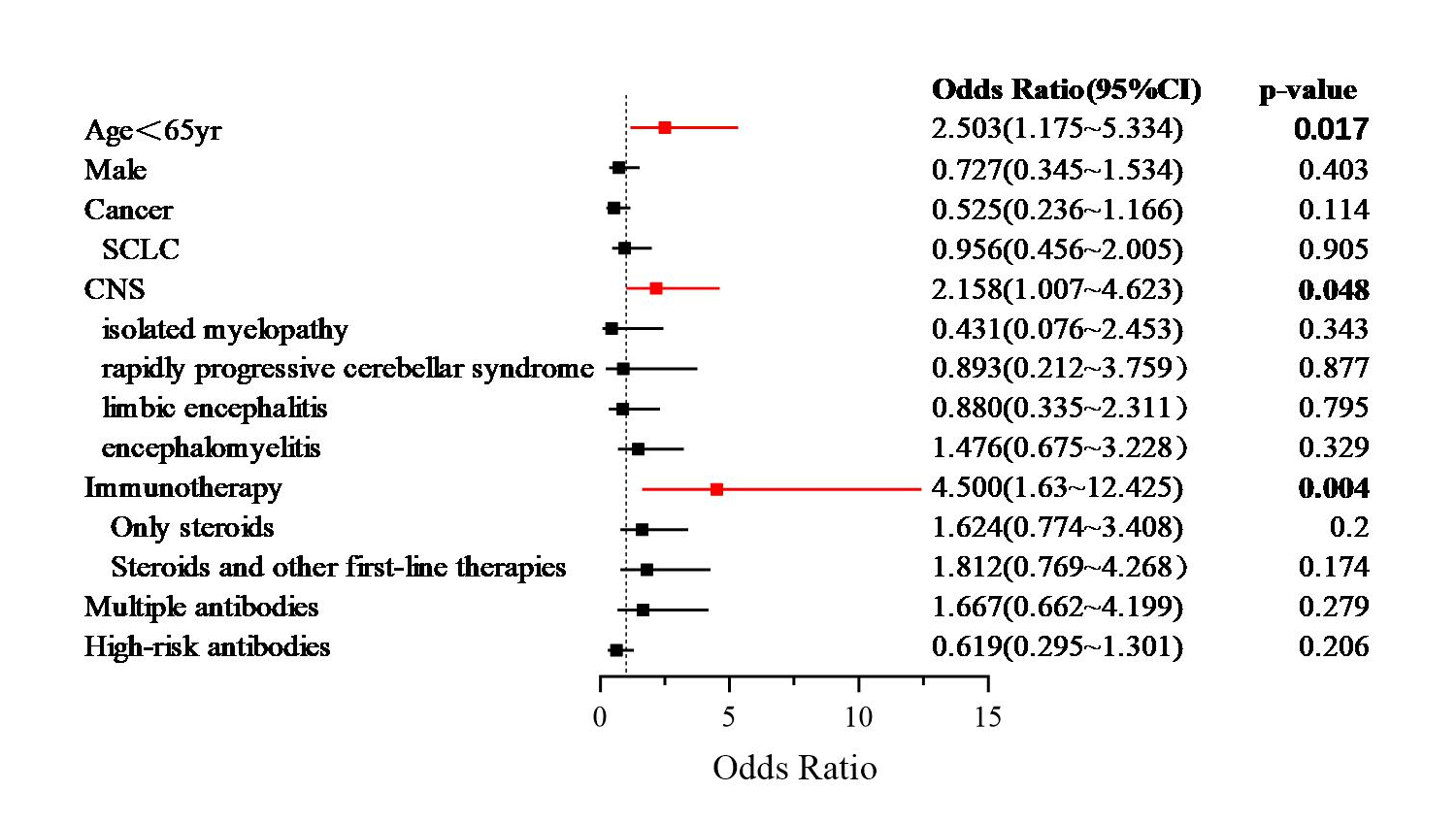

Supplement: Supplementary Figure 1 — Forest plot with univariate logistic regression predicting curative effect in patients at discharge. Error bars: 95% confidence interval. SCLC, small cell lung cancer; CNS, central nervous system. [file Image1.tif]
